# Meningothelial Cells React to Elevated Pressure and Oxidative Stress

**DOI:** 10.1371/journal.pone.0020142

**Published:** 2011-05-17

**Authors:** Xiaorong Xin, Bin Fan, Josef Flammer, Neil R. Miller, Gregor P. Jaggi, Hanspeter E. Killer, Peter Meyer, Albert Neutzner

**Affiliations:** 1 Department of Biomedicine, Ocular Pharmacology and Physiology and Department of Ophthalmology, University Hospital Basel, Basel, Switzerland; 2 Wilmer Ophthalmological Institute, Johns Hopkins Hospital, Baltimore, Maryland, United States of America; 3 Department of Ophthalmology, Kantonsspital Aarau, Aarau, Switzerland; Istituto Dermopatico dell'Immacolata, Italy

## Abstract

**Background:**

Meningothelial cells (MECs) are the cellular components of the meninges enveloping the brain. Although MECs are not fully understood, several functions of these cells have been described. The presence of desmosomes and tight junctions between MECs hints towards a barrier function protecting the brain. In addition, MECs perform endocytosis and, by the secretion of cytokines, are involved in immunological processes in the brain. However, little is known about the influence of pathological conditions on MEC function; e.g., during diseases associated with elevated intracranial pressure, hypoxia or increased oxidative stress.

**Methods:**

We studied the effect of elevated pressure, hypoxia, and oxidative stress on immortalized human as well as primary porcine MECs. We used MTS (3-(4,5-dimethylthiazol-2-yl)-5-(3-carboxymethoxyphenyl)-2-(4-sulfophenyl)-2H-tetrazolium) bioreduction assays to assess the proliferation of MECs in response to treatment and compared to untreated control cells. To assess endocytotic activity, the uptake of fluorescently labeled latex beads was analyzed by fluorescence microscopy.

**Results:**

We found that exposure of MECs to elevated pressure caused significant cellular proliferation and a dramatic decrease in endocytotic activity. In addition, mild oxidative stress severely inhibited endocytosis.

**Conclusion:**

Elevated pressure and oxidative stress impact MEC physiology and might therefore influence the microenvironment of the subarachnoid space and thus the cerebrospinal fluid within this compartment with potential negative impact on neuronal function.

## Introduction

The meninges cover and protect the brain and its “appendices”, the optic nerves. Whereas the dura mater forms the outer layer, the arachnoid and the pia mater form the inner layers of the meninges, with the pia mater facing the brain tissue. The space between the arachnoid and pia mater is the cerebrospinal fluid (CSF) filled subarachnoid space. The arachnoid, the pia mater as well as the arachnoid trabeculae and septae traversing the subarachnoid space are lined with meningothelial cells. Thus, these cells surround the CSF-filled subarachnoid space, providing a barrier between CSF and neuronal tissue on one side and between CSF and circulation on the other. As such, MECs share various features with other cell types with similar function such as vascular endothelial cells that form the blood-brain barrier. These features include the formation of tight cell-cell contacts such as desmosomes [1] and/or tight junctions [2] leading to a firm cellular layer that restricts free diffusion of substances between the CSF and the brain tissue. In addition, they were shown to actively produce and secret cytokines [3] and they are involved in the production of L-PGDS (lipocalin-type prostaglandin D2 synthase or β-trace protein), a multifunctional protein of the CSF [4], [5]. In addition, MECs are known to perform endocytosis [6]. However, MECs are still largely unexplored and their reaction to pathological conditions as well as a potential role during disease is not well understood.

Several diseases of the central nervous system, such as brain tumors, cranial vein occlusions, pseudotumor cerebri or meningitis, lead to increased intracranial pressure that sometimes is associated with oxidative stress or hypoxia [7], [8], [9]. This increase in pressure might have an influence on MECs through either mechanical stress or biochemical mechanisms mediated through an altered CSF compartment.

Using immortalized human as well as primary porcine MECs, we addressed the question of whether certain stress conditions such as elevated pressure and oxidative stress lead to changes in MEC proliferation or endocytotic activity.

## Results

### Pressure dependent proliferation rate

**Figure 1:**
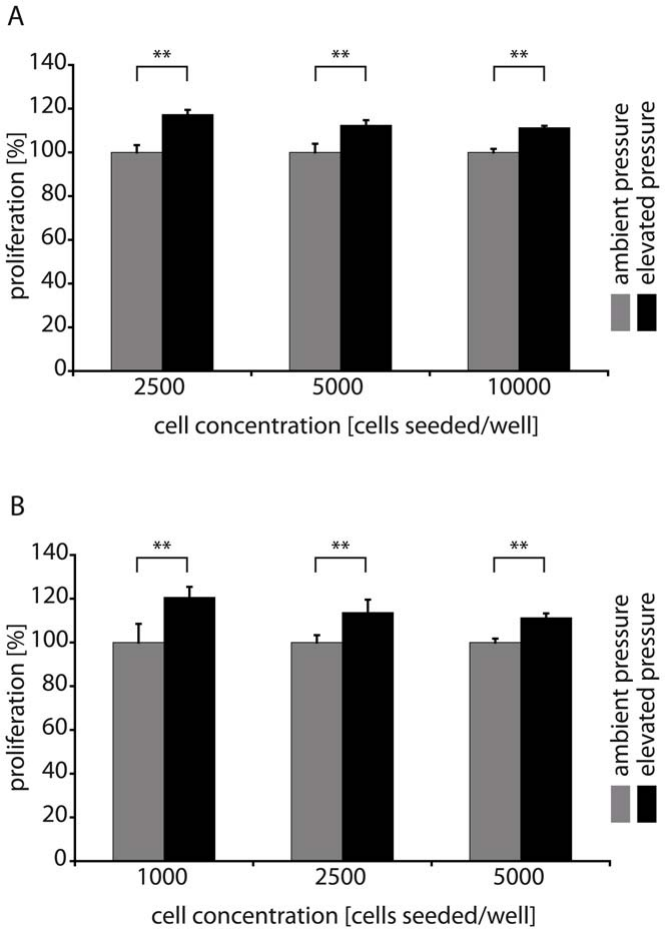
Biomechanical stress induces MEC proliferation. Proliferation of Ben-Men-I cells (A) and primary porcine MECs (B) exposed to elevated and ambient pressure for 2 days. Proliferation of Ben-Men-I cells and PMECs increased in all cell concentration groups that were exposed to elevated pressure compared to control cells cultivated under ambient pressure conditions (shown is a representative result of three independent experiments; error bars represent SD; Student's t-test: p<0.01, marked with ** for highly significant).

To study the effect of pressure on MECs, we exposed immortalized human as well as porcine MECs to elevated pressure and compared them with control cells incubated at ambient pressure, with ambient pressure meaning atmospheric pressure and elevated pressure meaning a pressure 30 mmHg above atmospheric pressure. We found that incubation of Ben-Men-I cells [10] for 2 days under 30 mmHg elevated pressure resulted in a 20% increase in their proliferation compared with cells incubated at ambient pressure as measured using MTS assays (Figure 1A). To exclude effects of gas-mixture composition and other chamber-related parameters, we performed the same experiment with cells inside the pressure chamber and gas application set to 0 mmHg additional pressure. We did not find a statistically significant difference between cells inside and outside the non-pressurized pressure chamber, consistent with the conclusion that additional pressure and not other effects of the pressure chamber setup caused the observed proliferation of the MECs (data not shown). To further establish the proliferation-enhancing effect of elevated pressure on MECs, we also treated primary porcine MECs [11] prepared from optic nerve sheaths (PMECs) with elevated pressure. As observed for immortalized Ben-Men-I cells, elevated pressure caused enhanced proliferation in PMECs as well (Figure 1B).

### Pressure impacts endocytosis by MECs

**Figure 2:**
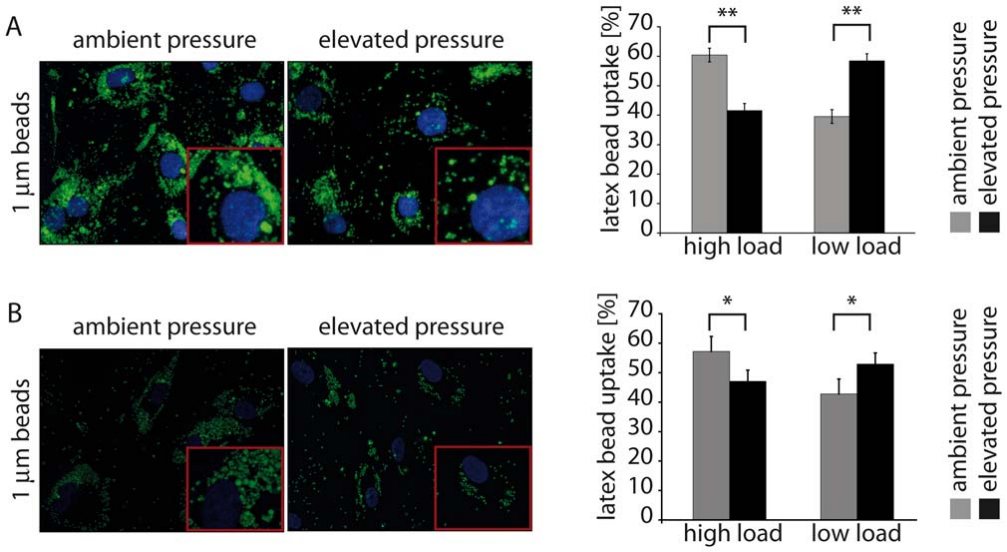
Endocytotic function of MECs is impacted by elevated pressure. Ben-Men-I cells (A) or PMECs (B) were treated with elevated pressure for two days and fluorescent latex beads were added to assess endocytotic activity by fluorescence microscopy (>100 cells scored/condition). Ben-Men-I cells (Student's t-test: p<0.01, marked with ** for highly significant) as well as PMECs (Student's t-test: p<0.05, marked with * for significant) showed a significant decrease in endocytotic activity after pressure treatment compared to control treated cells (shown is a representative result of three independent experiments; error bars represent SD).

To analyze endocytosis by MECs, the uptake of fluorescently-labeled latex particles was analyzed by fluorescence microscopy, scored visually, and the results compared with those of untreated control cells. Ben-Men-I cells (Figure 2A) and, to a somewhat lesser degree, PMECs (Figure 2B) exposed to elevated pressure lost their high endocytotic activity. Whereas about 60% of MECs exposed to ambient pressure displayed a high latex bead load, about 40% of cells exposed to high pressure displayed a high bead load.

### MEC proliferation and endocytosis under hypoxic conditions

**Figure 3:**
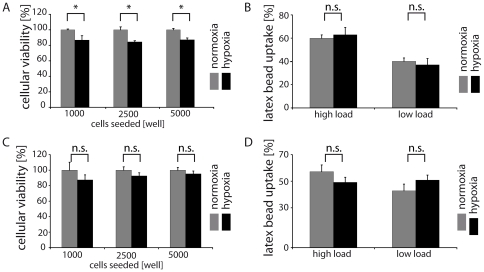
Hypoxia does not impact MEC function. Ben-Men-I cells and PMECs were incubated under limited oxygen conditions (1% O_2_) and compared to cells cultivated under normoxia. Cellular viability was only slightly impacted by this treatment in Ben-Men-I (A – representative result shown of three independent experiments; error bars represent SD; Student's t-test: p<0.05, marked with *) but not significantly in PMECs (C, marked with n.s. for not significant). Evaluation of endocytotic activity by scoring fluorescent latex bead uptake did not reveal a significant difference after hypoxia in Ben-Men-I cells (B, marked with n.s. for not significant) or PMECs (D, marked with n.s. for not significant).

After exposing Ben-Men-I cells and PMECs to hypoxic conditions, we found that cellular survival was slightly diminished by hypoxia in Ben-Men-I cells, whereas survival of PMECs was unaffected as assessed by MTS assay (Figure 3A and C). In addition, the endocytotic activity in both cell types (Figure 3B and D) was not significantly affected under these conditions.

### Oxidative stress impacts MEC proliferation and endocytosis

**Figure 4:**
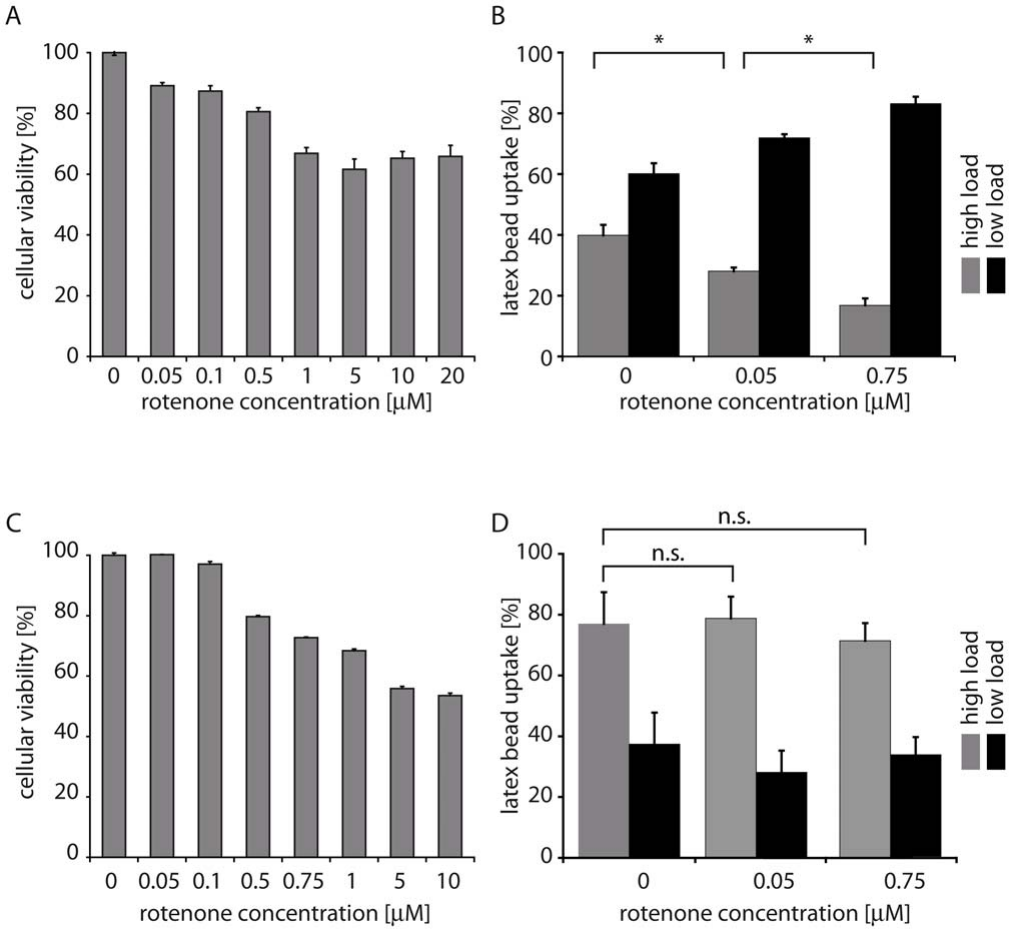
Oxidative stress impairs MEC function. Ben-Men-I cells (A; error bars represent +SEM; ANOVA p<0.01, Tukey's HSD post-hoc test see Figure S1A) (5×10^4^ cells/ml) or PMECs (C; error bars represent +SEM; ANOVA p<0.01, Tukey's HSD post-hoc test see Figure S1B) were treated with varying concentrations of the complex I inhibitor rotenone (0–20 µM) for 24 hours to induce oxidative stress. Oxidative damage resulting in decreased viability was measured using MTS. The impact of oxidative stress on MEC function in terms of endocytosis was analyzed by scoring fluorescent latex bead uptake following treatment with different concentrations (0, 0.05 and 0.75 µM) of rotenone. Intensity of latex beads engulfed by Ben-Men-I cells (B; >100 cells/condition; error bars represent SD of three independent counts; Student's t-test: p<0.05; marked with * for significant) or PMECs (D; >100 cells/condition; error bars represent SD of three independent counts; Student's t-test: p>0.05; marked with n.s. for not significant) decreases with increasing of rotenone concentration where Ben-Men-I cells display a high sensitivity towards rotenone with a reduction in endocytosis at low, non toxic rotenone concentrations while PMECs are more resistant to oxidative stress and maintain their rate of endocytosis.

To study the effect of oxidative stress on the proliferation and endocytosis of MECs, Ben-Men-I and PMECs were exposed to rotenone, an inhibitor of the mitochondrial electron transport chain complex I known to cause the production of reactive oxygen species [12]. Upon this treatment, MECs showed a dose-dependent loss of viability in response to rotenone compared with control cells. Although high concentrations of rotenone (20 µM) reduced the viability of Ben-Men-I cells to around 70% and of PMECs to around 50%, low concentrations (0.05 to 0.1 µM) had virtually no effect on cell proliferation. However, even concentrations of rotenone well below toxic levels (0.05 µM) caused a severe decline of endocytotic activity in Ben-Men-I cells (Figure 4B), whereas PMECs were less sensitive to rotenone treatment and maintained endocytosis under these conditions (Figure 4D).

## Discussion

The localization of MECs at the interface between CSF and neuronal tissue implies an important function for these cells in maintaining brain function; however, the role of MECs is far from being understood, and the cross-talk between MECs and the CSF compartment is not well explored. In this study, we examined whether or not conditions encountered by MECs in the course of certain diseases causing elevated intracranial pressure, hypoxic conditions or increased oxidative stress might influence the function of these cells.

As elevated pressure in the range of 30 to 50 mmHg was found to influence a variety of cellular function *in vivo* and *in vitro* [13], [14], [15] and is often associated with various brain diseases [7], [8], [9], we first asked if meningothelial cells react to this biomechanical stress. We focused on 30 mmHg as elevated pressure condition based on the difference between normal intracranial (around 10 mmHg) and severely elevated intracranial pressure (above 40 mmHg). We used ambient pressure as control treatment instead of a normal intracranial pressure of 10 mmHg, since the pressure difference and not the absolute pressure is most likely sensed by the cell with the our cultured cells adapted to ambient pressure.

We observed two distinct effects in response to elevated pressure: (A) increased proliferation of MECs and (B) reduced endocytosis both in immortalized human and primary porcine MECs. The fact that enhanced proliferation as well as reduced endocytosis was evident in two unrelated cell cultures from different organisms argues for a general effect of elevated pressure on MECs.

The observation of increased proliferation of MECs exposed to elevated pressure suggests an attempt by the cells to maintain integrity of the meninges in the face of mechanical stress. However, proliferation of MECs can become deleterious at sites of confined CSF space, such as the subarachnoid space of the optic nerve [16], as the width of the subarachnoid space here measures only about 0.3 mm and is further restricted through numerous trabeculae and septa. Thickening of the arachnoid or the pia mater in such a region through MEC proliferation might obstruct the flow of CSF and might, in effect, lead to CSF compartmentalization. It is conceivable that this, in turn, might negatively influence CSF composition and, therefore, might further harm MECs or even impact neuronal function. Interestingly, impairment of CSF dynamics has been reported in patients with papilledema [17], and significant proliferation of MECs has been reported in a post-mortem study in glaucoma patients [18], pointing to a pathophysiological role of these cells in a neurodegenerative disorder.

This impact of MECs on CSF might not be limited to the mechanical obstruction of CSF flow due to unwanted proliferation. Based on the observation that MECs are actively ingesting large quantities of particles, a role in clearing waste products from the CSF through endocytosis by MECs is conceivable. Our finding that elevated pressure decreased endocytosis suggests that such a CSF clearing function of MECs might be also decreased in diseases associated with elevated intracranial pressure. And since elevated pressure causes increased proliferation and down-regulates endocytosis, both proliferation and decreased endocytosis might cause synergistically diminished CSF turnover in affected areas. This decrease of CSF turnover might, in addition, be enhanced by accompanying oxidative stress. Oxidative stress in the CSF compartment is believed to be involved in the pathogenesis of various diseases including Alzheimer disease [19] and glaucoma [20]. How endocytosis is affected by oxidative stress in our meningothelial cell model remains unclear, especially since a difference in the reaction to oxidative stress between immortalized human and primary porcine MECs is noted that might be attributable to a different rotenone sensitivity of these cells; however, the amount of rotenone used to induce oxidative stress in MECs in our study was well below lethal levels as assessed by proliferation assays. We therefore consider a general toxic effect of rotenone unlikely and favor a more direct mechanism by which reactive oxygen species specifically interfere with the endocytotic machinery. Together with the above discussed MEC proliferation, the drop in endocytosis activity under oxidative stress conditions might additionally exacerbate problems in CSF maintenance. Although MEC proliferation might decrease the flow of CSF through certain areas, possibly causing waste product accumulation, diminished endocytosis not only due to increased pressure but also to oxidative stress might then under these conditions hamper waste removal by MECs. Interestingly and in contrast to pressure and oxidative stress, hypoxic conditions did not influence this potential waste removal mechanism in both analyzed cell models although immortalized human MECs showed a slight drop in proliferation while porcine MECs were not affected by low oxygen tension. These data point to a high resistance of MECs towards hypoxia and their ability to maintain CSF clearance under these conditions while elevated pressure as well as oxidative stress is less well tolerated.

Taken together, meningothelial cells provide important shielding function to the brain. This study sheds light on the interplay between MECs and their surroundings and points to possible connections between MEC function and pathological processes suggesting a more active role for these cells in maintaining brain function as previously appreciated.

## Materials and Methods

### Cell culture and treatment

Human WHO grade I meningioma-derived, hTERT-immortalized Ben-Men-I cells (DMSZ, Braunschweig) [10] were cultured in DMEM supplemented with 10% fetal calf serum, 2 mM L-glutamine, and 1 mM sodium pyruvate (Sigma-Aldrich, Buchs). Primary porcine meningothelial cells (PMECs) were cultured as previously described [11]. In short, PMECs were scraped from the inside of the optic nerve sheath from porcine eyes obtained fresh from the local slaughterhouse. After outgrowth, contaminating fibroblasts were removed using magnetic anti-fibroblast beads (Miltenyi, Bergisch Gladbach).

To induce oxidative stress, cells were treated with varying concentrations (0–20 µM) of the mitochondrial complex I inhibitor rotenone or with DMSO (Sigma-Aldrich, Buchs) as a vector control. Pressure treatment was performed in a Plexiglas box with two openings inside a normal cell culture incubator. One opening was connected to a pressurized gas cylinder (certified: 5% CO_2_, 21% O_2_, 74% N_2_) and the other opening was connected to a tube placed inside a 40 cm water column (equals 30 mmHg) to act as pressure regulator. Before entering the pressure chamber, the gas mixture was humidified and pre-warmed by slowly bubbling through a gas wash bottle kept inside the incubator. The gas flow was set to around one bubble per second as monitored at the tubing outlet in the water column.

For hypoxic conditions, cells were cultivated in an incubator under 1% oxygen and 5% CO_2_.

### Cell proliferation assay

Cells were seeded at indicated densities into 96 well plates and grown under the indicated conditions. To determine cellular viability, we incubated samples with [3-(4,5-dimethylthiazol-2-yl)-5-(3-carboxymethoxyphenyl)-2-(4-sulfophenyl)-2H-tetrazolium salt (MTS) (CellTiter 96 Aqueous One Solution Cell Proliferation Assay – Promega, Dübendorf) according to the manufacturer's instructions. Cell proliferation was calculated by A_treatment_/A_control_×100%, where A represents the absorbance recorded at 490 nm.

### Endocytosis assay

Cells were seeded at 60,000 cells/well into 12 well plates (Sarstedt, Sevelen) on top of a cover slip glass, grown in DMEM with 10% fetal calf serum, 2 mM L-glutamine, 1 mM sodium pyruvate (Sigma-Aldrich, Buchs) and treated for 1 day with the mitochondrial electron transport chain inhibitor rotenone at increasing concentrations (0–20 µM) to induce oxidative stress or in a specialized chamber at elevated and ambient pressure conditions for 2 days. Fluorescently-labeled latex beads (1 µm diameter, Sigma-Aldrich, Buchs) were added to the medium and the cells were incubated for another 24 hours, after which the cells were removed from the medium, fixed in 4% paraformaldehyde in PBS (Pierce) for 30 min, permeabilized with 0.15% Triton X-100 in PBS for 15 min, blocked with PBS containing 10% bovine serum albumin for 1 hour, and then washed with PBS. Cells were counterstained with DAPI for 3 minutes, mounted on glass slides, and observed using a fluorescence microscope (Olympus B×51, Japan) to determine the intensity of the uptake of latex beads by visual examination. Cells were categorized visually according to the amount of latex beads into “high load” for cells with a large amount of latex beads (up to 200 beads/cell) and into “low load” for cells with a small amount of beads inside the cytosol (around 20 beads/cells). Due to the large amount of ingested beads per cell, the beads were not counted individually, but were categorized by the observer.

### Statistical analysis

Data were analyzed by Student's t-test or ANOVA with Tukey's HSD (Honestly Significant Difference) post-hoc test. p-values<0.05 were considered significant and were marked with *, while p-values<0.01 were considered highly significant and were marked with **. No significant difference (p>0.05) was marked with n.s. for not significant. Error bars represent standard deviation (SD) unless stated otherwise.

## Supporting Information

Figure S1 **Statistical analysis of MEC sensitivity to rotenone treatment.** ANOVA analysis with Tukey's HSD post-hoc test for (A) Figure 4A or (B) Figure 4C. The analysis was performed using Statistica software.
(DOC)
