# Supplementary material for: Meningothelial Cells React to Elevated Pressure and Oxidative Stress
Source: PLoS One. 2011 May 17;6(5):e20142. doi: 10.1371/journal.pone.0020142 (PMC3096659; doi:10.1371/journal.pone.0020142)
Supplement: Figure S1 — Statistical analysis of MEC sensitivity to rotenone treatment. ANOVA analysis with Tukey's HSD post-hoc test for (A) Figure 4A or (B) Figure 4C. The analysis was performed using Statistica software. (DOC) [file pone.0020142.s001.doc]

**Figure S1**

**A**

|  | df | | MS | df | MS |  |  |  |  |
| --- | --- | --- | --- | --- | --- | --- | --- | --- | --- |
|  | Effect | | Effect | Error | Error | F | p-level |  |  |
|  | 7 | | 0.014783 | 16 | 0.000175 | 84.5164 | 1.93E-11 |  |  |
|  |  | |  |  |  |  |  |  |  |
|  |  | |  |  |  |  |  |  |  |
| Tukey HSD test | | | | |  |  |  |  |  |
| Probabilities for Post Hoc Test | | | |  |  |  |  |  |  |
|  | | | |  |  |  |  |  |  |
| μM rotenone | | 0 | 0.05 | 0.1 | 0.5 | 1 | 5 | 10 | 20 |
|  | | .4910003 | .4370003 | .4280003 | .3946670 | .3263337 | .3003337 | .3183337 | .3213337 |
| 0 | |  | 0.002673 | 0.000656 | 0.000175 | 0.000174 | 0.000174 | 0.000174 | 0.000174 |
| 0.05 | | 0.002673 |  | 0.988202 | 0.020957 | 0.000174 | 0.000174 | 0.000174 | 0.000174 |
| 0.1 | | 0.000656 | 0.988202 |  | 0.099389 | 0.000175 | 0.000174 | 0.000174 | 0.000174 |
| 0.5 | | 0.000175 | 0.020957 | 0.099389 |  | 0.00036 | 0.000175 | 0.000214 | 0.000249 |
| 1 | | 0.000174 | 0.000174 | 0.000175 | 0.00036 |  | 0.300867 | 0.994077 | 0.999701 |
| 5 | | 0.000174 | 0.000174 | 0.000174 | 0.000175 | 0.300867 |  | 0.70663 | 0.543076 |
| 10 | | 0.000174 | 0.000174 | 0.000174 | 0.000214 | 0.994077 | 0.70663 |  | 0.99999 |
| 20 | | 0.000174 | 0.000174 | 0.000174 | 0.000249 | 0.999701 | 0.543076 | 0.99999 |  |

B

|  | df | MS | df | MS |  |  |  |  |
| --- | --- | --- | --- | --- | --- | --- | --- | --- |
|  | Effect | Effect | Error | Error | F | p-level |  |  |
|  | 7 | 0.030427 | 16 | 7.59E-05 | 401.0129 | 9.33E-17 |  |  |
|  |  |  |  |  |  |  |  |  |
|  |  |  |  |  |  |  |  |  |
| Tukey HSD test; | | | |  |  |  |  |  |
| Probabilities for Post Hoc Tests | | |  |  |  |  |  |  |
|  | | |  |  |  |  |  |  |
| μM rotenone | 0 | 0.05 | 0.1 | 0.5 | 0.75 | 1 | 5 | 10 |
|  | .5333330 | .5343330 | .5179996 | .4259997 | .3893330 | .3666663 | .3003330 | .2883330 |
| 0 |  | 1 | 0.423627 | 0.000174 | 0.000174 | 0.000174 | 0.000174 | 0.000174 |
| 0.05 | 1 |  | 0.351924 | 0.000174 | 0.000174 | 0.000174 | 0.000174 | 0.000174 |
| 0.1 | 0.423627 | 0.351924 |  | 0.000174 | 0.000174 | 0.000174 | 0.000174 | 0.000174 |
| 0.5 | 0.000174 | 0.000174 | 0.000174 |  | 0.002016 | 0.000177 | 0.000174 | 0.000174 |
| 0.75 | 0.000174 | 0.000174 | 0.000174 | 0.002016 |  | 0.083076 | 0.000174 | 0.000174 |
| 1 | 0.000174 | 0.000174 | 0.000174 | 0.000177 | 0.083076 |  | 0.000175 | 0.000174 |
| 5 | 0.000174 | 0.000174 | 0.000174 | 0.000174 | 0.000174 | 0.000175 |  | 0.694943 |
| 10 | 0.000174 | 0.000174 | 0.000174 | 0.000174 | 0.000174 | 0.000174 | 0.694943 |  |
